# Supplementary material for: Hybrid Models and Biological Model Reduction with PyDSTool
Source: PLoS Comput Biol. 2012 Aug 9;8(8):e1002628. doi: 10.1371/journal.pcbi.1002628 (PMC3415397; doi:10.1371/journal.pcbi.1002628)
Supplement: Text S4 — Complete source code for the PyDSTool package (version 0.88.120504). Includes API documentation and help files linking to web pages. This file is identical to the current public release on Sourceforge.net. (ZIP) [file pcbi.1002628.s004.zip › PyDSTool/html/PyDSTool.Toolbox.fracdim-pysrc.html]

xml version="1.0" encoding="ascii"?


PyDSTool.Toolbox.fracdim


| Home | Trees | Indices | Help | PyDSTool |
| --- | --- | --- | --- | --- |

Package PyDSTool ::
Package Toolbox ::
Module fracdim

[hide private]

[frames] | no frames]

# Source Code for Module PyDSTool.Toolbox.fracdim

```
   1  """Fractal dimension estimates for analysis of datasets having rank 2 or 3.
 
   2  
 
   3  A journal publication concerning the use of these functions appears in
 
   4  IEEE Transactions on BioMedical Engineering, 2008.
 
   5  
 
   6  (c) 2005, 2006. Robert Clewley, John Guckenheimer.
 
   7  """ 
   8  
 
   9  from __future__ import division 
  10  from PyDSTool import * 
  11  from PyDSTool.common import _seq_types 
  12  from PyDSTool.Toolbox.data_analysis import * 
  13  from numpy import extract, mean, std, ndarray 
  14  from scipy.linalg import norm 
  15  from matplotlib.pylab import figure, plot, hold, polyfit, \
 
  16       bar, subplot, axes, title, xlabel, ylabel, draw, \
 
  17       getp, setp, gca, savefig, scatter, colorbar 
  18  from matplotlib import cm 
  19  from time import localtime 
  20  from copy import copy 
  21  
 
  22  __all__ = ['corr_dim', 'corr_dim_with_progress',
 
  23             'find_cover', 'find_outliers', 'get_cover_radii',
 
  24             'do_stats', 'nhd', 'timeseq', 'get_rV_curves',
 
  25             'get_filtered_ixs', 'plot_radius_distribution', 'filter_by_radius',
 
  26             'plot_dim_by_thickness', 'plot_thickness_by_dim', 'find_nhd',
 
  27             'prep_secant_figure', 'PD_E', 'slope_range', 'scatterplot_slopes',
 
  28             'scatter_histo', 'rescatter', 'sorted_by_slope', 'sorted_by_radius'] 
  29  
 
  30  # -----------------------------------------------------------------------------
 
  31  
 
  32  
 


33 -def corr_dim(data, which_norm=2):


34      """Correlation dimension of a data set.
 
  35      """ 
  36      npts = size(data,0) 
  37      d = zeros((npts*(npts-1)/2,), 'd')  # size of upper triangle (no diagonal) of m vs. m matrix 
  38      # max ix for each i in expression for d is (i-1)*(i-2)/2 + (i-2)
 
  39      for i in xrange(1,npts): 
  40          for j in xrange(i-1): 
  41              d[(i-1)*(i-2)/2+j] = norm(data[i]-data[j], which_norm) 
  42      d.sort() 
  43      logd = log(d).ravel() 
  44      logv = log(range(1,len(d)+1)) 
  45      nan_ixs = isfinite(logd) 
  46      logd = extract(nan_ixs, logd) 
  47      logv = extract(nan_ixs, logv) 
  48      return (logv, logd)

  49  
 
  50  
 


51 -def corr_dim_with_progress(data, inforate=10000, which_norm=2):


52      """Version of correlation dimension with text file progress dump,
 
  53      at a sample rate of 'inforate', because the calculation typically
 
  54      takes a long time.
 
  55      """ 
  56      npts = size(data,0) 
  57      dsize = npts*(npts-1)/2 
  58      d = zeros((dsize,), 'd')  # size of upper triangle (no diagonal) of m vs. m matrix 
  59      # max ix for each i in expression for d is (i-1)*(i-2)/2 + (i-2)
 
  60      for i in xrange(1,npts): 
  61          for j in xrange(i-1): 
  62              ix = (i-1)*(i-2)/2+j 
  63              if mod(ix,inforate) == 0: 
  64                  dump_progress(ix, dsize) 
  65              d[ix] = norm(data[i]-data[j]) 
  66      d.sort() 
  67      logd = log(d).ravel() 
  68      logv = log(range(1,len(d)+1)) 
  69      nan_ixs = isfinite(logd) 
  70      logd = extract(nan_ixs, logd) 
  71      logv = extract(nan_ixs, logv) 
  72      return (logv, logd)

  73  
 
  74  
 


75 -def timeseq(time, covering, refpt):


76      """Extract time sequence from a covering""" 
  77      ts = time.take(covering[refpt][3], 0) 
  78      print "time sequence min = %f, max = %f"%(min(ts),max(ts)) 
  79      return ts

  80  
 
  81  
 
  82  
 
  83  #### Version 2 of regression residual method
 
  84  #### This one starts at sqrt(N)
 


85 -def find_cover(data, remain_tol=5,
 
  86                 step=None, initref=None, wlo=1, whi=1,
 
  87                 check_large_nhds=True, large_nhd=300,
 
  88                 min_nhd_size=50, min_ix=25,
 
  89                 max_res=1.0, max_std=0.2, quiet=True):


90      """
 
  91      OUTPUTS:
 
  92  
 
  93       covering is a dict:
 
  94          reference point -> (dim, low radius, high radius, covered_indices)
 
  95       covered is a dict:
 
  96          point -> reference points whose neighbourhoods include it
 
  97      """ 
  98      covering = {} 
  99      covered = {}.fromkeys(range(len(data)),None) 
 100      not_covered = covered.keys() 
 101      if remain_tol > len(data): 
 102          remain_tol = 0 
 103      makerefpt_ixs = [] 
 104      if initref is not None: 
 105          refptix = initref 
 106      else: 
 107          refptix = 0   # initial value 
 108      N = size(data,0) 
 109      start_ix = int(round(sqrt(N))) 
 110      print "N = ", N, "start ix = ", start_ix 
 111      logv_raw = log(range(1,N+1)) 
 112      done = False 
 113      unsuccessful = [] 
 114      if step is None: 
 115          ixstep = max([int(around(len(data)/2000.)),1]) 
 116      elif step > 0: 
 117          if step < 1: 
 118              # treat as %age of # data points
 
 119              ixstep = int(math.ceil(len(d)*step)) 
 120              if not quiet: 
 121                  print "Using index step %i out of %i points"%(ixstep, len(d)) 
 122          else: 
 123              ixstep = step 
 124      else: 
 125          raise ValueError("Invalid step argument") 
 126      while not done: 
 127          if not quiet: 
 128              print "\n*************\nCalculating distances and point-wise dimension around pt %i ..."%refptix 
 129          logv, logd, d, d_inv_dict = log_distances_with_D(data, refptix, logv_raw) 
 130          d_inv_keys = sortedDictKeys(d_inv_dict) 
 131          d_inv_vals = sortedDictValues(d_inv_dict) 
 132          d_inv = Pointset(indepvararray=d_inv_keys, indepvartype=Float,
 
 133                           coordarray=d_inv_vals, coordtype=Int, tolerance=5e-5) 
 134          if not quiet: 
 135              print "Finding self-consistent neighbourhood", 
 136          # search from min_ix -> end, increasing hiix from loix+1 until dimensionality
 
 137          # (slope) stabilizes to within an absolute tolerance and does not grow larger
 
 138          # than a different tolerance
 
 139          err=-1 
 140          len_logd = len(logd) 
 141          old_dim = None 
 142          hiix = start_ix 
 143          if not quiet: 
 144              print "Start ix = ", start_ix 
 145          loix=start_ix-2 
 146          nhd_too_small = False 
 147          ref_dim = -1 
 148          all_res = [] 
 149          in_zone = False 
 150          # Grow West!
 
 151          while not in_zone and loix > min_ix+ixstep: 
 152              loix -= ixstep 
 153              pfit=polyfit(logd[loix:hiix],logv[loix:hiix],1) 
 154              dim = pfit[0] 
 155              residual = get_linear_regression_residual(pfit, logd[loix:hiix],logv[loix:hiix],
 
 156                                                        weight='') 
 157              all_res.append(residual) 
 158              if not quiet and mod(hiix,50) == 0: 
 159                  print ".", 
 160              if old_dim is not None: 
 161                  sd = std(all_res) 
 162  ##                if residual > max_res:
 
 163  ##                    in_zone = True
 
 164                  if sd > max_std: 
 165                      in_zone = True 
 166              old_dim = dim 
 167          # Grow East!
 
 168          all_res = [] #[all_res[-1]] 
 169          not_done = True 
 170          while not_done and hiix < len_logd-1-ixstep: 
 171              hiix += ixstep 
 172              # do a straight-line best fit
 
 173              pfit=polyfit(logd[loix:hiix],logv[loix:hiix],1) 
 174              dim = pfit[0] 
 175              residual = get_linear_regression_residual(pfit, logd[loix:hiix],logv[loix:hiix],
 
 176                                                        weight='lo', w=wlo) 
 177              all_res.append(residual) 
 178              if not quiet: 
 179                  print "(%i,%i)"%(loix,hiix), 
 180                  print "dim=%.4f, residual = %.4f"%(dim,residual) 
 181              if not quiet and mod(hiix,50) == 0: 
 182                  print ".", 
 183              try: 
 184                  sd = std(all_res) 
 185              except: 
 186                  sd = 0 
 187  ##            if residual > max_res:
 
 188  ##                if not quiet:
 
 189  ##                    print "residual > max_res"
 
 190  ##                not_done = False
 
 191              if sd > max_std: 
 192                  if not quiet: 
 193                      print "residuals s.d. > max_std" 
 194                  not_done = False 
 195              old_dim = dim 
 196          nhd_too_small = hiix-loix < min_nhd_size 
 197          if not quiet: 
 198              print "nhd_too_small = ", nhd_too_small 
 199          if nhd_too_small: 
 200              if not quiet: 
 201                  print "Neighbourhood too small. Moving to a different reference point..." 
 202              unsuccessful.append(refptix) 
 203          else: 
 204              if not quiet: 
 205                  print "\nDimension = %f"%dim 
 206                  print "Found best fit line from relative ix %i to %i (radius %f)"%(loix, hiix, d[hiix]) 
 207              # consolidate results in terms of global index positions in data
 
 208              covered_ixs = [d_inv(d[ix])[0] for ix in range(loix, hiix+1)] 
 209              covered_ixs.append(refptix) 
 210              # if nhd very large, recheck one in every 100 points because ptwise
 
 211              #  dim estimate may occasionally vary between points in that nhd.
 
 212              if hiix-loix > large_nhd and check_large_nhds: 
 213                  num_recheck = int(around((hiix-loix)/large_nhd)) 
 214                  for i in range(num_recheck): 
 215                      ix = random.randrange(0, hiix-loix) 
 216                      if ix not in makerefpt_ixs and ix != refptix: 
 217                          makerefpt_ixs.append(ix) 
 218              covering[refptix] = (dim, d[loix], d[hiix], covered_ixs) 
 219              for ix in covered_ixs: 
 220                  if covered[ix] is None: 
 221                      covered[ix] = [refptix] 
 222                  else: 
 223                      covered[ix].append(refptix) 
 224              not_covered = remain(not_covered, covered_ixs) 
 225              # only assert this as a consistency check the first time through the loop
 
 226  ##            assert len(not_covered) + len(covered_ixs) + 1 == lendat
 
 227          # find new ref pt in not_covered and repeat
 
 228          num_uncovered = len(not_covered) 
 229  ##        if not quiet:
 
 230          print "%i points left to cover"%(num_uncovered+len(makerefpt_ixs)) 
 231          if num_uncovered < remain_tol: 
 232              if len(makerefpt_ixs) == 0: 
 233                  done = True 
 234              else: 
 235                  # check these ref pts that were part of large nhds
 
 236                  refptix = makerefpt_ixs.pop() 
 237                  check_large_nhds = False   # switch this off now! 
 238          else: 
 239              pick_unsuccessful = 0 
 240              while pick_unsuccessful < num_uncovered and pick_unsuccessful >= 0: 
 241                  refptix = not_covered[random.randrange(0,num_uncovered)] 
 242                  if refptix in unsuccessful: 
 243                      pick_unsuccessful += 1 
 244                  else: 
 245                      pick_unsuccessful = -1 
 246              if pick_unsuccessful == num_uncovered: 
 247                  done = True 
 248      return (covered, covering)

 249  
 
 250  
 
 251  
 


252 -def find_nhd(data, tol_up=.2, tol_down=0.05, quiet=True, max_res=0.2, max_std=0.1,
 
 253               step=None, initref=None, min_ix=10, min_nhd_size=20,
 
 254               max_radius_ratio=1.5):


255      """Find a single neighbourhood around a reference point.""" 
 256      covering = {} 
 257      covered = {}.fromkeys(range(len(data)),None) 
 258      if initref is not None: 
 259          refptix = initref 
 260      else: 
 261          refptix = 0   # initial value 
 262      logv_raw = log(range(1,size(data,0)+1)) 
 263      if not quiet: 
 264          print "\n*************\nCalculating distances and point-wise dimension around pt %i ..."%refptix 
 265          doplot = 1 
 266      else: 
 267          doplot = 0 
 268      logv, logd, d, d_inv_dict = log_distances_with_D(data, refptix, logv_raw) 
 269      d_inv_keys = sortedDictKeys(d_inv_dict) 
 270      d_inv_vals = sortedDictValues(d_inv_dict) 
 271      d_inv = Pointset(indepvararray=d_inv_keys, indepvartype=Float,
 
 272                       coordarray=d_inv_vals, coordtype=Int, tolerance=5e-5) 
 273      if not quiet: 
 274          print "Finding self-consistent neighbourhood", 
 275      # search from min_ix -> end, increasing hiix from loix+1 until dimensionality
 
 276      # (slope) stabilizes to within an absolute tolerance and does not grow larger
 
 277      # than a different tolerance
 
 278      err=-1 
 279      len_logd = len(logd) 
 280      old_dim = None 
 281      loix=min_ix 
 282      hiix=loix+1 
 283      nhd_too_small = (len_logd < min_ix+10) 
 284      if step is None: 
 285          ixstep = max([int(around(len(data)/2000.)),1]) 
 286      elif step > 0: 
 287          if step < 1: 
 288              # treat as %age of # data points
 
 289              ixstep = int(math.ceil(len(d)*step)) 
 290              if not quiet: 
 291                  print "Using index step %i out of %i points"%(ixstep, len(d)) 
 292          else: 
 293              ixstep = step 
 294      else: 
 295          raise ValueError("Invalid step argument") 
 296      if not quiet: 
 297          plot(logd,logv) 
 298      not_done = not nhd_too_small 
 299      in_zone = False 
 300      ref_dim = -1 
 301      log_max_radius_ratio = log(max_radius_ratio) 
 302      all_res = [] 
 303      while not_done and hiix < len_logd-1-ixstep: 
 304          hiix += ixstep 
 305          # do a straight-line best fit
 
 306          pfit = polyfit(logd[loix:hiix],logv[loix:hiix],1) 
 307          dim = pfit[0] 
 308          residual = get_linear_regression_residual(pfit, logd[loix:hiix],logv[loix:hiix]) 
 309  ##        if hiix - loix > min_nhd_size:
 
 310          all_res.append(residual) 
 311          if not quiet: 
 312              print "Dim = %.3f in [%i,%i], Residual = %.4f "%(dim,loix,hiix,residual), 
 313              plot([logd[loix],logd[hiix]],[logd[loix]*pfit[0]+pfit[1],logd[hiix]*pfit[0]+pfit[1]]) 
 314  ##            if mod(hiix,50) == 0:
 
 315  ##                print ".",
 
 316          if old_dim is not None: 
 317  ##            err = abs(dim-old_dim)/dim
 
 318              sd = std(all_res) 
 319              if not quiet: 
 320                  print "S.d. = %.4f "%sd 
 321              if residual > max_res: 
 322                  print "residual > max_res" 
 323                  not_done = False 
 324              if sd > max_std: 
 325                  print "residuals s.d. > max_std" 
 326                  not_done = False 
 327  ##            if err > max_err and hiix-loix > min_nhd_size/4:
 
 328  ##                if not quiet:
 
 329  ##                    print "\nMaxed out successive difference in dimension estimate"
 
 330  ##                    print " ... current dim = %.3f, old dim = %.3f, error = %.3f"%(dim,old_dim,err)
 
 331  ##                not_done = False
 
 332  ##                continue
 
 333  ##            if in_zone:
 
 334  ##                zone_err = abs(dim-ref_dim)/ref_dim
 
 335  ##                if in_zone and zone_err > tol_up:
 
 336  ##                    not_done = False  # end neighbourhood growth
 
 337  ##                if logd[hiix]-logd[loix] > log_max_radius_ratio:
 
 338  ##                    not_done = False  # end neighbourhood growth
 
 339  ##                    if not quiet:
 
 340  ##                        print "Radius too large: %.4f/%.4f"%(exp(logd[hiix]),exp(logd[loix]))
 
 341  ##            elif err < tol_down:
 
 342  ##                in_zone = True
 
 343  ##                ref_dim = dim
 
 344  ##                if not quiet:
 
 345  ##                    print " - entered zone at ix", hiix, " - with ref dim ", ref_dim
 
 346          old_residual = residual 
 347          old_dim = dim 
 348      nhd_too_small = nhd_too_small or hiix-loix < min_nhd_size 
 349      if nhd_too_small: 
 350          print "Neighbourhood too small. Try a different starting index or a new reference point ..." 
 351          print "Dim found over ixs [%i, %i] = %.4f"%(loix,hiix,dim) 
 352          raise RuntimeError 
 353      else: 
 354          if not quiet: 
 355              print "\nDimension = %f"%dim 
 356              print "Found best fit line from relative ix %i to %i (radius %f)"%(loix, hiix, d[hiix]) 
 357          # consolidate results in terms of global index positions in data
 
 358          covered_ixs = [d_inv(d[ix])[0] for ix in range(loix, hiix+1)] 
 359          covered_ixs.append(refptix) 
 360          covering[refptix] = (dim, d[loix], d[hiix], covered_ixs) 
 361          for ix in covered_ixs: 
 362              if covered[ix] is None: 
 363                  covered[ix] = [refptix] 
 364              else: 
 365                  covered[ix].append(refptix) 
 366      return (covered, covering)

 367  
 
 368  
 
 369  
 


370 -def do_stats(covering, covered, maxD, bin_width=1,
 
 371               fignum=1, num_panels=4, s_frac=0.5,
 
 372               nhd_size_thresh=None, nhd_max_plot_size=None, minD=None,
 
 373               D_estimate_method="absolute", weight_by_size=False,
 
 374               force_xaxis_limits=False, radius_yaxis_limits=None,
 
 375               save=''):


376      """Produces statistics of pointwise dimension estimates of a data set,
 
 377      including a histogram of the distribution of neighbourhood dimensions.
 
 378  
 
 379      R. Clewley, June 2006.
 
 380  
 
 381      INPUTS:
 
 382  
 
 383      maxD sets the maximum dimension to cater for.
 
 384  
 
 385      bin_width selects the width of bins for dimensions (default 1).
 
 386  
 
 387      fignum forces a particular figure number to be used for display.
 
 388          (Use fignum=0 to suppress plotting of the results and just return
 
 389           the statistics.)
 
 390  
 
 391      num_panels argument must be 2 (for just nhd size and bin histo) or
 
 392         4 (also include min and max nhd radius).
 
 393  
 
 394      s_frac selects the fraction (or multiple) of the standard deviation of
 
 395         neighbourhood sizes to use as the cutoff for the "bulk" of the data
 
 396         set, in order to avoid including large outliers in determining
 
 397         nhd_size_threshold (default 0.5).
 
 398  
 
 399      nhd_size_threshold selects the smallest neighbourhood size to be
 
 400         included in histogram of neighbourhood dimensionalities
 
 401         (default None => automatic selection, i.e., the mean of the first
 
 402          s_frac of the neighbourhood sizes' standard deviation).
 
 403         Set to zero to prevent it being used or plotted.
 
 404  
 
 405      nhd_max_plot_size selects the y-axis limit on the plot of neighbourhood
 
 406         sizes (default None => automatic selection).
 
 407  
 
 408      D_estimate_method can be 'absolute' (uses cutoff of size = 1 to estimate D
 
 409         in histogram tail), or 'relative' (uses a cutoff = %age of peak binned
 
 410         neighbourhoods)
 
 411  
 
 412      force_xaxis_limits (Boolean) determines whether x-axis limits should be
 
 413         forced to be the value of the (minD,maxD) parameters.
 
 414  
 
 415      minD sets the minimum dimension to cater for (default to auto-choice).
 
 416  
 
 417      radius_yaxis_limits (pair) sets the r_min and r_max y-axis upper limits,
 
 418         respectively.
 
 419  
 
 420      save (string) sets a filename to use for saving the figure in available
 
 421         formats (so don't include a file extension).
 
 422  
 
 423  
 
 424      OUTPUTS:
 
 425  
 
 426      d_stats: dictionary {"D_tail": estimated D from tail,
 
 427                           "D_mean": histo mean,
 
 428                           "D_std": histo std dev}
 
 429  
 
 430      dbins: Pointset of D bins
 
 431  
 
 432      cover_by_dimix: dictionary of covers by index of dimensions binned
 
 433         (i.e., [0, bin_width, 2*bin_width, ..., maxD+1])
 
 434  
 
 435      cover_by_size: list of neighbourhood coverings ordered by their size
 
 436  
 
 437      cbu: array indicating how many points in overlap between neighbourhoods
 
 438          (array index corresponds to a number of points shared,
 
 439           value = number of nhds with that number of points shared)
 
 440  
 
 441      ixs: list of indices of neighbourhoods of size > nhd_size_threshold
 
 442  
 
 443      (integral: internal diagnostic feature)
 
 444      """ 
 445      dx = zeros((len(covering),1),'f') 
 446      ly = zeros((len(covering),1),'f') 
 447      rloy = zeros((len(covering),1),'f') 
 448      rhiy = zeros((len(covering),1),'f') 
 449      drange = arange(0, maxD+1, bin_width, 'd').tolist() 
 450      dbins = data_bins('D', drange) 
 451      cover_by_dimix = {}.fromkeys(range(len(drange))) 
 452      for dix in range(len(drange)): 
 453          cover_by_dimix[dix] = [] 
 454      cover_by_size_dict = {}.fromkeys(range(len(covered)+1)) 
 455      ix = 0 
 456      integral = 0 
 457      largest = 0 
 458      try: 
 459          for p, (d, rlo, rhi, l) in covering.iteritems(): 
 460              dx[ix] = d 
 461              rloy[ix] = rlo 
 462              rhiy[ix] = rhi 
 463              lenl = len(l) 
 464              if lenl > largest: 
 465                  largest = lenl 
 466              try: 
 467                  cover_by_size_dict[lenl].append(p) 
 468              except AttributeError: 
 469                  cover_by_size_dict[lenl] = [p] 
 470              ly[ix] = lenl 
 471              integral += lenl 
 472              ix += 1 
 473      except ValueError: 
 474          # No max radius information available!
 
 475          # compatible with old version of covering that does not return rhi
 
 476          for p, (d, rlo, l) in covering.iteritems(): 
 477              dx[ix] = d 
 478              rloy[ix] = rlo 
 479              rhiy[ix] = 0 
 480              lenl = len(l) 
 481              if lenl > largest: 
 482                  largest = lenl 
 483              try: 
 484                  cover_by_size_dict[lenl].append(p) 
 485              except AttributeError: 
 486                  cover_by_size_dict[lenl] = [p] 
 487              ly[ix] = lenl 
 488              integral += lenl 
 489              ix += 1 
 490      print "\nNeighbourhood statistics:" 
 491      print "There are %i neighbourhoods to this covering"%len(covering) 
 492      print "Largest neighbourhood has %i points"%largest 
 493      cover_by_size = [di for di in cover_by_size_dict.iteritems() if di[1] is not None] 
 494      cover_by_size.sort() 
 495      csizes = [c[0] for c in cover_by_size] 
 496      if nhd_size_thresh is None: 
 497          s = std(csizes) 
 498          sm = s_frac*s 
 499          m = mean(csizes) 
 500          print "Std. dev. of cover_by_size =", s 
 501          print "Max size found =", max(csizes) 
 502          print "Mean size found =", m 
 503  ##        # find largest index of set of covering nhds such that its size
 
 504  ##        # is smaller than s_frac% of the std deviation of the sizes
 
 505  ##        for i in range(len(cover_by_size)):
 
 506  ##            if csizes[i] > sm:
 
 507  ##                break
 
 508  ##        try:
 
 509  ##            nhd_size_thresh = mean(csizes[:i])
 
 510  ##        except ZeroDivisionError:
 
 511  ##            nhd_size_thresh = 0
 
 512  ##        print "N'hood size threshold used = mean(cover_by_sizes restricted to %.3f of 1st std. dev.)"%s_frac
 
 513  ##        print "                           =", nhd_size_thresh
 
 514          nhd_size_thresh = m-sm 
 515          print "N'hood size threshold used = mean - %.3f of std. dev."%s_frac 
 516          print "                           =", nhd_size_thresh 
 517      else: 
 518          if nhd_size_thresh > 0: 
 519              print "N'hood size threshold set by user =", nhd_size_thresh 
 520      if nhd_max_plot_size is None: 
 521  ##        # 2 * original set sizes mean
 
 522  ##        try:
 
 523  ##            nhd_max_plot_size = 2*mean(csizes)
 
 524  ##        except ZeroDivisionError:
 
 525  ##            nhd_max_plot_size = largest
 
 526  ##        print "Using max plot nhd size = 2*mean(cover_by_sizes) =", nhd_max_plot_size
 
 527          nhd_max_plot_size = largest 
 528          print "Using max plot nhd size of largest neighbourhood found =", nhd_max_plot_size 
 529      else: 
 530          print "Using max plot nhd size set by user =", nhd_max_plot_size 
 531      # reverse so that largest first for returning to user
 
 532      cover_by_size.reverse() 
 533      filtered_ixs = [] 
 534      try: 
 535          for p, (d, rlo, rhi, l) in covering.iteritems(): 
 536              if d <= maxD: 
 537                  dix = dbins.resolve_bin_index(d) 
 538                  if len(l) > nhd_size_thresh: 
 539                      if weight_by_size: 
 540                          inc = len(l)/largest 
 541                      else: 
 542                          inc = 1 
 543                      dbins.increment(d, inc) 
 544                      filtered_ixs.append(p) 
 545                  cover_by_dimix[dix].append(p) 
 546      except ValueError: 
 547          # compatible with old version of covering that does not return rhi
 
 548          for p, (d, rlo, l) in covering.iteritems(): 
 549              if d <= maxD: 
 550                  dix = dbins.resolve_bin_index(d) 
 551                  if len(l) > nhd_size_thresh: 
 552                      if weight_by_size: 
 553                          inc = len(l)/largest 
 554                      else: 
 555                          inc = 1 
 556                      dbins.increment(d, inc) 
 557                      filtered_ixs.append(p) 
 558                  cover_by_dimix[dix].append(p) 
 559      # decide on single dimension estimate
 
 560      ks=dbins.midpoints 
 561      d_est = 0   # initial value 
 562      if D_estimate_method == "absolute": 
 563          # based on largest D > 1 in
 
 564          # tail of dbins histogram (assumes unimodal)
 
 565          if len(covering) < 3: 
 566              dbin_thresh = 0 
 567          else: 
 568              dbin_thresh = 1 
 569          for k in ks: 
 570              n = dbins(k) 
 571              if d_est > 0 and n == 0: 
 572                  # stop before a second spike might occur
 
 573                  break 
 574              if n > dbin_thresh: 
 575                  d_est = k 
 576      elif D_estimate_method == "relative": 
 577          peak = 0 
 578          last_peak = 0 
 579          for k in ks: 
 580              n = dbins(k) 
 581              if n > peak and n > 1: 
 582                  peak = n 
 583                  last_peak = n 
 584              if d_est > 0 and n == 0: 
 585                  # stop before a second spike might occur
 
 586                  break 
 587              if peak > 0: 
 588                  # only applies if peak already found
 
 589                  if n > .2*last_peak: 
 590                      # discard bins if smaller than 20% of last peak
 
 591                      d_est = k 
 592                      # only change last peak record if new one is actually smaller!
 
 593                      last_peak = n 
 594      else: 
 595          raise ValueError("Invalid dimension estimation method name") 
 596      if fignum > 0: 
 597          fontmath = args(fontsize=22,fontname='Times') 
 598          fonttext = args(fontsize=18,fontname='Times') 
 599          # make plot
 
 600          print "Plotting histograms to figure", fignum 
 601          assert num_panels in [2,4], "num_panels argument must be 2 or 4" 
 602          figure(fignum) 
 603          if num_panels == 4: 
 604              subplot(2,2,1) 
 605              plot(dx, rhiy, 'ro') 
 606              glines = getp(gca(), 'xticklines')+getp(gca(), 'yticklines') 
 607              setp(glines, 'linewidth', 2) 
 608  ##            title('nhd max radius')
 
 609              xlabel(r'$\rm{Dimension}$', fonttext) 
 610              ylabel(r'$r_{max}$', fontmath) 
 611          if num_panels == 4: 
 612              subplot(2,2,2) 
 613          else: 
 614              subplot(2,1,1) 
 615          plot(dx, ly, 'ko') 
 616          glines = getp(gca(), 'xticklines')+getp(gca(), 'yticklines') 
 617          setp(glines, 'linewidth', 2) 
 618          dmin, dmax = figure(fignum).axes[0].get_xlim() 
 619          if dmax > maxD or force_xaxis_limits: 
 620              dmax = maxD 
 621          if dmin < minD or force_xaxis_limits: 
 622              if minD is not None: 
 623                  dmin = minD 
 624          if nhd_size_thresh > 0: 
 625              plot([dmin,dmax],[nhd_size_thresh,nhd_size_thresh],'k--',linewidth=1.2) 
 626  ##        title('size')
 
 627          xlabel(r'$\rm{Dimension}$', fonttext) 
 628          ylabel(r'$\rm{N-hood \ size}$', fonttext) 
 629          if num_panels == 4: 
 630              subplot(2,2,3) 
 631              plot(dx, rloy, 'go') 
 632              glines = getp(gca(), 'xticklines')+getp(gca(), 'yticklines') 
 633              setp(glines, 'linewidth', 2) 
 634  ##            title('minimum radius')
 
 635              xlabel(r'$\rm{Dimension}$', fonttext) 
 636              ylabel(r'$r_{min}$', fontmath) 
 637          if num_panels == 4: 
 638              subplot(2,2,4) 
 639          else: 
 640              subplot(2,1,2) 
 641          dbx = dbins.midpoints.tolist() 
 642          dby = dbins.values() 
 643          bar(dbx, dby, color='b', width=dbx[1]-dbx[0]) 
 644          for panel in range(num_panels): 
 645              figure(fignum).axes[panel].set_xlim([dmin,dmax]) 
 646  ##        title('number binned')
 
 647          xlabel(r'$\rm{Dimension}$', fonttext) 
 648          ylabel(r'$\rm{Number \ binned}$',fonttext) 
 649      # Calculate overlap info:
 
 650      # how many times does a point appear in the neighbourhoods?
 
 651      # assumes will not be more than 100 times!
 
 652      cbins = zeros((100,),'i') 
 653      max_used = 0 
 654      for clist in covered.itervalues(): 
 655          try: 
 656              n = len(clist) 
 657          except TypeError: 
 658              # a point that was not included in a covering
 
 659              n = 0 
 660          if n > max_used: 
 661              max_used = n 
 662          if n < maxD+1: 
 663              cbins[n] = cbins[n] + 1 
 664  ##    cran = range(max_used+1)
 
 665      cbu = cbins[0:max_used+1] 
 666      if fignum > 0: 
 667  ##        subplot(2,2,4)
 
 668  ##        bar(cran,cbu)
 
 669  ##        title('nhd overlap')
 
 670  ##        xlabel('# pts in common')
 
 671  ##        ylabel('# nhds')
 
 672          if num_panels == 4: 
 673              figure(fignum).axes[0].set_position([0.1, 0.55, 0.37, 0.35]) 
 674              figure(fignum).axes[1].set_position([0.57, 0.55, 0.37, 0.35]) 
 675              figure(fignum).axes[2].set_position([0.1, 0.09, 0.37, 0.35]) 
 676              figure(fignum).axes[3].set_position([0.57, 0.09, 0.37, 0.35]) 
 677              figure(fignum).axes[1].set_ylim([0,nhd_max_plot_size]) 
 678              if radius_yaxis_limits is not None: 
 679                  assert radius_yaxis_limits[1] >= radius_yaxis_limits[0], \
 
 680                         "Specify radius y-axis limits in increasing order" 
 681                  ylo1 = figure(fignum).axes[0].get_ylim()[0] 
 682                  ylo0 = figure(fignum).axes[2].get_ylim()[0] 
 683                  figure(fignum).axes[0].set_ylim([ylo1,radius_yaxis_limits[1]]) 
 684                  figure(fignum).axes[2].set_ylim([ylo0,radius_yaxis_limits[0]]) 
 685          else: 
 686              figure(fignum).axes[0].set_position([0.125, 0.57, 0.775, 0.364]) 
 687              figure(fignum).axes[1].set_position([0.125, 0.08, 0.775, 0.364]) 
 688              figure(fignum).axes[0].set_ylim([0,nhd_max_plot_size]) 
 689          draw() 
 690          if save != '': 
 691              savefig(save+'.png') 
 692              savefig(save+'.svg') 
 693              savefig(save+'.eps') 
 694      # don't assert the following if re-checking points in large nhds (the default)
 
 695      #assert sum([i*cbins[i] for i in cran]) == integral
 
 696      tail_ix = dbins.resolve_bin_index(d_est) 
 697      if isinstance(tail_ix, tuple): 
 698          tail_ix = tail_ix[1] 
 699      try: 
 700          m = dbins[:tail_ix].mean() 
 701          s = dbins[:tail_ix].std() 
 702      except: 
 703          print "Tail found at index %i and D = %.3f"%(tail_ix,d_est) 
 704          print " -- problem computing mean and std dev for this mode" 
 705          print " so calculating for whole data set" 
 706          m = dbins.mean() 
 707          s = dbins.std() 
 708      print "Estimate dimension to be (to resolution of histogram bin width):", d_est 
 709      print "  with histogram mean D = %.5f and std dev D = %.5f"%(m, s) 
 710      return {"D_tail": d_est, "D_mean": m, "D_std": s}, \
 
 711             dbins, cover_by_dimix, cover_by_size, cbu, filtered_ixs, integral

 712  
 
 713  
 


714 -def get_filtered_ixs(cover_by_dimix):


715      """Returns the list of indices in covering for which the neighbourhood
 
 716      size was larger than the threshold for dimension binning.""" 
 717      fixs=[] 
 718      for p, ixlist in cover_by_dimix.iteritems(): 
 719          fixs.extend(ixlist) 
 720      fixs.sort() 
 721      return fixs

 722  
 
 723  
 


724 -def find_outliers(data, plotdata, cluster_dim, cluster_width, max_central=50):


725      outliers = [] 
 726      central = [] 
 727      assert cluster_width > 0 and cluster_dim - cluster_width > 1 
 728      for i, (dlo, dhi, refix) in enumerate(plotdata): 
 729          refix = int(refix) 
 730          if dlo < 1: 
 731              outliers.append(refix) 
 732          if dlo > cluster_dim-cluster_width and dlo < cluster_dim+cluster_width: 
 733              if len(central) < max_central: 
 734                  central.append(refix) 
 735      num_central = len(central) 
 736      central_data = array([data[cix] for cix in central]) 
 737      if max_central > 0: 
 738          c_mean = mean(central_data,0) 
 739      else: 
 740          c_mean = central_data[0] 
 741      do = zeros((len(outliers),),float) 
 742      dc = zeros((num_central,),float) 
 743      for i, oix in enumerate(outliers): 
 744          do[i]=norm(c_mean-data[oix]) 
 745      for i, cdata in enumerate(central_data): 
 746          dc[i]=norm(c_mean-cdata) 
 747      ds = [] 
 748      for i in xrange(len(data)): 
 749          if i not in outliers: 
 750              ds.append(norm(data[i]-data[outliers[0]])) 
 751      ds = array(ds) 
 752      if num_central > 1: 
 753          mean_dc = mean(dc) 
 754          std_dc = std(dc) 
 755      else: 
 756          mean_dc = dc[0] 
 757          std_dc = 0 
 758      #print "Found %i outliers and %i central points"%(len(outliers),num_central)
 
 759      return (mean(do), std(do), mean_dc, std_dc, mean(ds), std(ds), do)

 760  
 
 761  
 


762 -def get_cover_radii(covering, ixs=None, method='divide'):


763      """Return the radius of a covering""" 
 764      if method == 'divide': 
 765          rfun = lambda rh, rl: rh/rl 
 766      elif method == 'subtract': 
 767          rfun = lambda rh, rl: rh-rl 
 768      radii = {} 
 769      if ixs is None: 
 770          for p, (d, rlo, rhi, l) in covering.iteritems(): 
 771              radii[p] = rfun(rhi,rlo) 
 772      else: 
 773          for p, (d, rlo, rhi, l) in covering.iteritems(): 
 774              if p in ixs: 
 775                  radii[p] = rfun(rhi,rlo) 
 776      return radii

 777  
 
 778  
 


779 -def filter_by_radius(covering, radii, rlo=None, rhi=None):


780      """Returns list of filtered (index, radius) pairs and corresponding
 
 781      sub-coverings' dimensions""" 
 782      if rhi is None: 
 783          assert rlo is not None 
 784          test = lambda r: r > rlo 
 785      else: 
 786          if rlo is None: 
 787              test = lambda r: r < rhi 
 788          else: 
 789              test = lambda r: r > rlo and r < rhi 
 790      rfilt = [(ix,r) for (ix, r) in radii.iteritems() if test(r)] 
 791      return rfilt, [covering[rinfo[0]][0] for rinfo in rfilt]

 792  
 
 793  
 


794 -def plot_radius_distribution(radii, max_radius, radius_bin_width):


795      """Expects radii dictionary, as returned by get_cover_radii.
 
 796      Returns figure handle.""" 
 797      rads = array(radii.values()) 
 798      dbins = data_bins('r', arange(0, max_radius+radius_bin_width, radius_bin_width)) 
 799      dbins.bin(rads) 
 800      dbx, dby = dbins.to_array() 
 801      f = figure() 
 802      bar(dbx,dby,width=rbin) 
 803      title('Distribution of nhd radii') 
 804      xlabel('r') 
 805      ylabel('#') 
 806      return f

 807  
 
 808  
 


809 -def plot_dim_by_thickness(covering, radii, rwidth=0, plotstyle='k.'):


810      """Plot of dimensions of neighbourhoods with thickness r, using
 
 811      binned data of bin width rwidth. r is defined as the neighbourhood's
 
 812      r_max - r_min.
 
 813  
 
 814      If rwidth=0 (default) then no data binning is done.
 
 815      Returns figure handle.""" 
 816      rlo, rhi = extent(radii.values()) 
 817      assert rlo > 0 and rhi > rlo 
 818      f=figure() 
 819      if rwidth > 0: 
 820          assert rwidth < rhi-rlo 
 821          for r in arange(0, rhi+rwidth, rwidth): 
 822              rs,ds=filter_by_radius(covering, radii, r, r+rwidth) 
 823              if ds != []: 
 824                  plot([r], ds, plotstyle) 
 825          title('Distribution of dimension by radius bin (width '+str(rwidth)+')') 
 826      else: 
 827          rs,ds=filter_by_radius(covering, radii, 0, rhi) 
 828          if ds != []: 
 829              plot([r[1] for r in rs], ds, plotstyle) 
 830  ##        title('Distribution of dimension by radius')
 
 831      xlabel(r'$r_{max} \ - \ r_{min}$',fontsize=22) 
 832      ylabel(r'$\rm{Dimension}$',fontsize=20) 
 833      return f

 834  
 
 835  
 


836 -def plot_thickness_by_dim(covering, radii, plotstyle='k.'):


837      """Plot of thickness of neighbourhoods as a function of their
 
 838      estimated dimension. r is defined as the neighbourhood's
 
 839      r_max - r_min.
 
 840  
 
 841      Returns figure handle.""" 
 842      rlo, rhi = extent(radii.values()) 
 843      assert rlo > 0 and rhi > rlo 
 844      f = figure() 
 845      rs, ds = filter_by_radius(covering, radii, 0, rhi) 
 846      if ds != []: 
 847          plot(ds, [r[1] for r in rs], plotstyle) 
 848      ylabel(r'$r_{max} \ - \ r_{min}$',fontsize=22) 
 849      xlabel(r'$\rm{Dimension}$',fontsize=20) 
 850      return f

 851  
 
 852  
 


853 -def nhd(data, covering, refpt):


854      return data.take(covering[refpt][3], 0)

 855  
 
 856  # ---------------------------------------------------------------------------
 
 857  
 
 858  
 


859 -def get_rV_curves(data, refpts, doplot=True, show_seq=False):


860      """Get r-V curves for pointwise dimension estimate of data""" 
 861      if doplot: 
 862          figure() 
 863      rV = {} 
 864      for refpt in refpts: 
 865          if show_seq: 
 866              logv, logd, ixs_sorted = log_distances(data, refpt, doplot,
 
 867                                                     return_ixs=True) 
 868          else: 
 869              logv, logd = log_distances(data, refpt, doplot,
 
 870                                                     return_ixs=False) 
 871          if show_seq: 
 872              # get points that are not in increasing or decreasing sequence
 
 873              notinseq = out_of_seq(ixs_sorted) 
 874              inseq = remain(ixs_sorted, notinseq) 
 875          else: 
 876              notinseq = [] 
 877              inseq = [] 
 878          rV[refpt] = (logv, logd, inseq, notinseq) 
 879      return rV

 880  
 
 881  
 


882 -def make_secant_fig_dict(items):


883      return {
 
 884          'Dim': items[0],
 
 885          'refpt': int(items[1]),
 
 886          'r_min_minslope': items[2],
 
 887          'r_min_maxslope': items[3],
 
 888          'index': int(items[4])
 
 889          }

 890  
 


891 -def prep_secant_figure(data, plotdata, ssorted, radii, ixdata, sstats,
 
 892                         start_ix, stop_ix, Delta, fignum, save='',
 
 893                         show_seq=False):


894      sms=sstats['min_slope'] 
 895      res_info = {'min_slope':{}, 'max_slope': {}} 
 896      keyslopes_min=[sms['max'], sms['min'], sms['mean'], sms['mean']+sms['std']] 
 897      if sms['mean']-sms['std'] > 0: 
 898          keyslopes_min.append(sms['mean']-sms['std']) 
 899      ixs_min = [int(i) for i in find_from_sorted(plotdata[:,0], keyslopes_min)] 
 900      refpts_min = [ixdata[ix][1] for ix in ixs_min] 
 901      rm=res_info['min_slope'] 
 902      rm['max'] = make_secant_fig_dict([sms['max'],
 
 903                                    refpts_min[0]]+radii[ixs_min[0]].tolist()) 
 904      rm['min'] = make_secant_fig_dict([sms['min'],
 
 905                                    refpts_min[1]]+radii[ixs_min[1]].tolist()) 
 906      rm['mean'] = make_secant_fig_dict([sms['mean'],
 
 907                                     refpts_min[2]]+radii[ixs_min[2]].tolist()) 
 908      rm['mean_plus_std'] = make_secant_fig_dict([sms['mean']+sms['std'], \
 
 909                                      refpts_min[3]]+radii[ixs_min[3]].tolist()) 
 910      if sms['mean']-sms['std'] > 0: 
 911          rm['mean_minus_std'] = make_secant_fig_dict([sms['mean']-sms['std'], \
 
 912                                      refpts_min[4]]+radii[ixs_min[4]].tolist()) 
 913  
 
 914      sms=sstats['max_slope'] 
 915      keyslopes_max=[sms['max'], sms['min'], sms['mean'], sms['mean']+sms['std']] 
 916      if sms['mean']-sms['std'] > 0: 
 917          keyslopes_max.append(sms['mean']-sms['std']) 
 918      ixs_max = [int(i) for i in find_from_sorted(plotdata[:,1], keyslopes_max)] 
 919      refpts_max = [ixdata[ix][1] for ix in ixs_max] 
 920      rm=res_info['max_slope'] 
 921      rm['max'] = make_secant_fig_dict([sms['max'],
 
 922                                    refpts_max[0]]+radii[ixs_max[0]].tolist()) 
 923      rm['min'] = make_secant_fig_dict([sms['min'],
 
 924                                    refpts_max[1]]+radii[ixs_max[1]].tolist()) 
 925      rm['mean'] = make_secant_fig_dict([sms['mean'],
 
 926                                     refpts_max[2]]+radii[ixs_max[2]].tolist()) 
 927      rm['mean_plus_std'] = make_secant_fig_dict([sms['mean']+sms['std'], \
 
 928                                      refpts_max[3]]+radii[ixs_max[3]].tolist()) 
 929      if sms['mean']-sms['std'] > 0: 
 930          rm['mean_minus_std'] = make_secant_fig_dict([sms['mean']-sms['std'], \
 
 931                                      refpts_max[4]]+radii[ixs_max[4]].tolist()) 
 932  
 
 933      rV=get_rV_curves(data, refpts_min+refpts_max, False, show_seq) 
 934      res_info['min_slope']['refpts'] = refpts_min 
 935      res_info['max_slope']['refpts'] = refpts_max 
 936      res_info['rV'] = rV 
 937      cols_min=['b','r','y']  # min_slope [max, min, mean] 
 938      cols_max=['c','m','g']  # max_slope [max, min, mean] 
 939      figure(fignum) 
 940      subplot(1,2,1) 
 941      xlim=[Inf,-Inf] 
 942      ylim=[Inf,-Inf] 
 943      for i in [0,1,2]: 
 944          logv, logd, inseq, notinseq = rV[refpts_min[i]] 
 945          plot(logd, logv, cols_min[i], linewidth=2) 
 946          if show_seq: 
 947              if len(inseq) > len(notinseq): 
 948                  # plot not in seq points in red
 
 949                  plot(logd[notinseq], logv[notinseq], 'r^') 
 950              else: 
 951                  # plot seq points in green
 
 952                  plot(logd[inseq], logv[inseq], 'gv') 
 953          minlogd = min(logd) 
 954          maxlogd = max(logd) 
 955          minlogv = min(logv) 
 956          maxlogv = max(logv) 
 957          if minlogd < xlim[0]: 
 958              xlim[0] = minlogd 
 959          if maxlogd > xlim[1]: 
 960              xlim[1] = maxlogd 
 961          if minlogv < ylim[0]: 
 962              ylim[0] = minlogv 
 963          if maxlogv > ylim[1]: 
 964              ylim[1] = maxlogv 
 965          logv, logd, inseq, notinseq = rV[refpts_max[i]] 
 966          plot(logd, logv, cols_max[i], linewidth=2) 
 967          if show_seq: 
 968              if len(inseq) > len(notinseq): 
 969                  # plot not in seq points in red
 
 970                  plot(logd[notinseq], logv[notinseq], 'r^') 
 971              else: 
 972                  # plot seq points in green
 
 973                  plot(logd[inseq], logv[inseq], 'gv') 
 974          minlogd = min(logd) 
 975          maxlogd = max(logd) 
 976          minlogv = min(logv) 
 977          maxlogv = max(logv) 
 978          if minlogd < xlim[0]: 
 979              xlim[0] = minlogd 
 980          if maxlogd > xlim[1]: 
 981              xlim[1] = maxlogd 
 982          if minlogv < ylim[0]: 
 983              ylim[0] = minlogv 
 984          if maxlogv > ylim[1]: 
 985              ylim[1] = maxlogv 
 986      # plot line marking where start_ix'th point lies
 
 987      plot(xlim, [logv[start_ix], logv[start_ix]], 'k--', linewidth=2) 
 988      # if stop_ix < N, plot line marking where stop_ix'th point lies
 
 989      if stop_ix < len(data): 
 990          plot(xlim, [logv[stop_ix], logv[stop_ix]], 'k--', linewidth=2) 
 991      # New loop, to ensure markers are on top of all lines:
 
 992      # Mark r_min and r_max for secant of both max and min slope,
 
 993      # for each r-V curve plotted
 
 994      for i in [0,1,2]: 
 995          logv, logd, inseq, notinseq = rV[refpts_min[i]] 
 996          loix_min = ixdata[ixs_min[i]][0][0] 
 997          hiix_min = find(logv, logv[loix_min]+log(Delta)) 
 998          plot([logd[loix_min]], [logv[loix_min]], cols_min[i]+'o',
 
 999               markersize=10) 
1000          plot([logd[hiix_min]], [logv[hiix_min]], cols_min[i]+'o',
 
1001               markersize=10) 
1002          loix_min = ixdata[ixs_min[i]][0][1] 
1003          hiix_min = find(logv, logv[loix_min]+log(Delta)) 
1004          plot([logd[loix_min]], [logv[loix_min]], cols_min[i]+'s',
 
1005               markersize=10) 
1006          plot([logd[hiix_min]], [logv[hiix_min]], cols_min[i]+'s',
 
1007               markersize=10) 
1008          logv, logd, inseq, notinseq = rV[refpts_max[i]] 
1009          loix_max = ixdata[ixs_max[i]][0][0] 
1010          hiix_max = find(logv, logv[loix_max]+log(Delta)) 
1011          plot([logd[loix_max]], [logv[loix_max]], cols_max[i]+'o',
 
1012               markersize=10) 
1013          plot([logd[hiix_max]], [logv[hiix_max]], cols_max[i]+'o',
 
1014               markersize=10) 
1015          loix_max = ixdata[ixs_max[i]][0][1] 
1016          hiix_max = find(logv, logv[loix_max]+log(Delta)) 
1017          plot([logd[loix_max]], [logv[loix_max]], cols_max[i]+'s',
 
1018               markersize=10) 
1019          plot([logd[hiix_max]], [logv[hiix_max]], cols_max[i]+'s',
 
1020               markersize=10) 
1021      # plot bar for Delta
 
1022      bar_x = 0.1*(xlim[1]-xlim[0])+xlim[0] 
1023      bar_y = 0.5*(ylim[1]-ylim[0])+ylim[0] 
1024      plot([bar_x, bar_x],[bar_y, bar_y+log(Delta)],'k-',linewidth=10) 
1025      # labels
 
1026      fontmath_h = args(fontsize=22,fontname='Times',
 
1027                        verticalalignment='top') 
1028      fontmath_v = args(fontsize=22,fontname='Times',
 
1029                        horizontalalignment='right') 
1030      xlabel(r'$\rm{log \ } r_k$',fontmath_h) 
1031      ylabel(r'$\rm{log \ } k$',fontmath_v) 
1032      subplot(1,2,2) 
1033      rescatter(plotdata, array([0.2,0.2,0.2]), newfigure=False) 
1034      # replot selected points
 
1035      for i in [0,1,2]: 
1036          rescatter(array([plotdata[ixs_min[i]]]), cols_min[i],
 
1037                    marker='o', marker_size=150, newfigure=False) 
1038          rescatter(array([plotdata[ixs_max[i]]]), cols_max[i],
 
1039                    marker='s', marker_size=225, newfigure=False) 
1040      figure(fignum).axes[0].set_xlim(xlim) 
1041      figure(fignum).axes[0].set_ylim(ylim) 
1042      figure(fignum).axes[0].set_position([0.07, 0.11, 0.41, 0.85]) 
1043      figure(fignum).axes[1].set_position([0.54, 0.11, 0.41, 0.85]) 
1044      figure(fignum).set_figwidth(14.5) 
1045      draw() 
1046      if save != '': 
1047          figure(fignum).savefig(save+'.png') 
1048          figure(fignum).savefig(save+'.svg') 
1049          figure(fignum).savefig(save+'.eps') 
1050      return res_info

1051  
 
1052  
 


1053 -def slope_range(data, refptix, step=2, Delta=10, startix=10, stopix=None,
 
1054                  doplot=False, maxD=Inf):


1055      """Slope range by secants over a range of log(Delta) ball volumes""" 
1056      N=size(data,0) 
1057      assert Delta > 1 
1058      assert N > Delta*startix 
1059      logv_raw = log(range(1,N+1)) 
1060      logv, logd, d, d_inv_dict = log_distances_with_D(data, refptix, logv_raw) 
1061      hi_slope = 0 
1062      lo_slope = 1e10 
1063      loix = startix 
1064      hiix = loix*Delta 
1065      if stopix is None: 
1066          stopix = N 
1067      assert stopix <= N 
1068      assert hiix < stopix 
1069      logDelta = log(Delta) 
1070      slope_by_loix = {} 
1071      while True: 
1072          # slope by secant
 
1073          slope = logDelta/(logd[hiix]-logd[loix]) 
1074          if slope > hi_slope and slope < maxD: 
1075              hi_slope = slope 
1076              hi_slope_ix = loix 
1077          if slope < lo_slope and slope < maxD: 
1078              lo_slope = slope 
1079              lo_slope_ix = loix 
1080          slope_by_loix[loix] = slope 
1081          loix += step 
1082          hiix = Delta*loix 
1083          if hiix >= stopix: 
1084              break 
1085      if doplot: 
1086          plot(sortedDictValues(slope_by_loix)) 
1087      return (slope_by_loix, lo_slope, lo_slope_ix,
 
1088              hi_slope, hi_slope_ix, logv, logd)

1089  
 
1090  
 
1091  
 


1092 -def scatterplot_slopes(data, num_samples=None, startix=10, stopix=None,
 
1093                         Delta=10, marker='o', marker_size=40, cmap=cm.hot,
 
1094                         color_source=None, step=5, fignum=None, maxD=Inf):


1095      """fignum = 0 switches off plotting (just return statistics)""" 
1096      if num_samples is None: 
1097          num_samples = len(data)/5 
1098          range_step = 5 
1099      else: 
1100          range_step = int(len(data)/num_samples) 
1101      if range_step > 0: 
1102          refpts = arange(0,len(data),range_step,'i') 
1103      else: 
1104          raise "too many sample points specified" 
1105      num_refpts = len(refpts) 
1106      plotdata = zeros((num_refpts,3),Float) 
1107      ixdata = {} 
1108      # colors indicate either min or max radii
 
1109      colors = zeros((num_refpts,3),Float) 
1110      if color_source == 'lo': 
1111          cix=0 
1112          title_str = 'min' 
1113      elif color_source == 'hi': 
1114          cix=1 
1115          title_str = 'max' 
1116      elif color_source is None: 
1117          title_str = '' 
1118          col = array([0.3,0.3,0.3]) 
1119      else: 
1120          raise "invalid color source" 
1121      print "Expect %i dots when finished:"%int(num_refpts/50) 
1122      for i in range(num_refpts): 
1123          if mod(i,50) == 49: 
1124              print ".", 
1125          slope_by_loix, lo, loix, hi, hiix, logv, logd = slope_range(data,
 
1126                              refpts[i], startix=startix, stopix=stopix,
 
1127                              Delta=Delta, step=step, maxD=maxD) 
1128          plotdata[i,:] = array([lo,hi,refpts[i]]) 
1129          ixdata[i] = [(loix,hiix),refpts[i]] 
1130          colors[i,:] = array([exp(logd[loix]),exp(logd[hiix]),refpts[i]]) 
1131      if fignum is None: 
1132          figure() 
1133          dofig = True 
1134      elif fignum == 0: 
1135          dofig = False 
1136      else: 
1137          figure(fignum) 
1138          dofig = True 
1139      if dofig: 
1140          if color_source is None: 
1141              handle=scatter(plotdata[:,0],plotdata[:,1],marker=marker,c=col,
 
1142                      s=marker_size) 
1143          else: 
1144              handle=scatter(plotdata[:,0],plotdata[:,1],marker=marker,c=colors[:,cix],
 
1145                      cmap=cmap,s=marker_size) 
1146              colorbar() 
1147          fonttext = args(fontsize=20,fontname='Times') 
1148          xlabel(r'$\rm{min \ slope}$',fonttext) 
1149          ylabel(r'$\rm{max \ slope}$',fonttext) 
1150          if title_str != '': 
1151              title('Color-coded by r_min of '+title_str+' slope') 
1152      else: 
1153          handle=None 
1154      s=(sorted_by_slope(plotdata,0),sorted_by_slope(plotdata,1)) 
1155      if color_source is None: 
1156          c = () 
1157      else: 
1158          c=(sorted_by_radius(colors,0),sorted_by_radius(colors,1)) 
1159      stats = {'min_slope': {}, 'max_slope': {}} 
1160      stats['min_slope']['mean']=mean(plotdata[:,0]) 
1161      stats['max_slope']['mean']=mean(plotdata[:,1]) 
1162      stats['min_slope']['std']=std(plotdata[:,0]) 
1163      stats['max_slope']['std']=std(plotdata[:,1]) 
1164      stats['min_slope']['min']=min(plotdata[:,0]) 
1165      stats['max_slope']['min']=min(plotdata[:,1]) 
1166      stats['min_slope']['max']=max(plotdata[:,0]) 
1167      stats['max_slope']['max']=max(plotdata[:,1]) 
1168      print "\n" 
1169      return (plotdata, s, colors, c, ixdata, stats, handle)

1170  
 
1171  
 


1172 -def sorted_by_slope(plotdata, select=0):


1173      """select: 0 = rmin, 1 = rmax""" 
1174      ixs = argsort(plotdata, 0)[:,select] 
1175      return zip(take(plotdata, ixs, 0)[:,select], ixs)

1176  
 


1177 -def sorted_by_radius(colors, select=0):


1178      """select: 0 = rmin, 1 = rmax""" 
1179      ixs = argsort(colors, 0)[:,select] 
1180      return zip(take(colors, ixs, 0)[:,select], ixs)

1181  
 


1182 -def find_from_sorted(x, v, next_largest=1, indices=None):


1183      if isinstance(v, _seq_types): 
1184          a=array(x) 
1185          res = [find(a, y, next_largest, indices) for y in v] 
1186          return res 
1187      else: 
1188          return find(array(x), v, next_largest, indices)

1189  
 
1190  
 


1191 -def rescatter(plotdata, colors, color_source=None, marker='o', marker_size=40,
 
1192                cmap=cm.hot, newfigure=True):


1193      if color_source == 'lo': 
1194          cix=0 
1195          title_str = 'min' 
1196      elif color_source == 'hi': 
1197          cix=1 
1198          title_str = 'max' 
1199      elif color_source is None: 
1200          title_str = '' 
1201          if not isinstance(colors, str): 
1202              assert shape(colors) == (3,) 
1203      else: 
1204          raise "invalid color source" 
1205      if newfigure: 
1206          figure() 
1207      if color_source is None: 
1208          handle=scatter(plotdata[:,0],plotdata[:,1],marker=marker,
 
1209                  c=colors,s=marker_size) 
1210      else: 
1211          handle=scatter(plotdata[:,0],plotdata[:,1],marker=marker,
 
1212                  c=colors[:,cix],cmap=cmap,s=marker_size) 
1213          colorbar() 
1214      fonttext_v = args(fontsize=22,fontname='Times',
 
1215                      horizontalalignment='right') 
1216      fonttext_h = args(fontsize=22,fontname='Times',
 
1217                      verticalalignment='top') 
1218      xlabel(r'$\rm{min \ slope}$',fonttext_h) 
1219      ylabel(r'$\rm{max \ slope}$',fonttext_v) 
1220      if title_str != '': 
1221          title('Color-coded by r_min of '+title_str+' slope') 
1222      return handle

1223  
 
1224  
 


1225 -def scatter_histo(plotdata,select=1,bin_width=1,maxD=None,
 
1226                    xlim=None,ylim=None):


1227      ds = array(plotdata[:,select]) 
1228      ds.shape = (len(plotdata),) 
1229      if maxD is None: 
1230          maxD = int(ceil(max(ds[:,0]))) 
1231      drange = arange(0, maxD+1+bin_width, bin_width, typecode='d') 
1232      dbins = data_bins('D', drange) 
1233      dbins.bin(ds) 
1234      dbx, dby = dbins.to_array() 
1235      f=figure() 
1236      b=bar(dbx, dby, color='b', width=dbx[1]-dbx[0]) 
1237      if xlim is not None: 
1238          f.axes[0].set_xlim(xlim) 
1239          draw() 
1240      if ylim is not None: 
1241          f.axes[0].set_ylim(ylim) 
1242          draw()

1243  
 
1244  
 


1245 -def PD_E(a, secfig=5, verbose=False, saveplot=True, force=False):


1246      """Pointwise dimension estimation (PD-E) using secants of r-V curves.
 
1247      See:
 
1248  
 
1249      J. Guckenheimer, "Dimension Estimates for Attractors",
 
1250      Contemporary Mathematics, Vol. 28, 1984.
 
1251  
 
1252      R. Clewley, J. Guckenheimer, F. Valero-Cuevas, "Estimating effective
 
1253      degrees of freedom in motor control", IEEE Transactions in Biomedical
 
1254      Engineering, 2007.
 
1255      """ 
1256      pde_name = 'PD_E-'+a.name 
1257      if 'N' in a.keys(): 
1258          if 'stopix' in a.keys(): 
1259              if a.stopix is not None: 
1260                  a.stopix = int(round(0.7*a.N)) 
1261          else: 
1262              a.stopix = int(round(0.7*a.N)) 
1263          a.num_samples = int(round(0.2*a.N)) 
1264          a.step = int(round(0.005*a.N)) 
1265      else: 
1266          a.N = None 
1267          if 'stopix' not in a.keys(): 
1268              a.stopix = None 
1269      try: 
1270          if force: 
1271              raise ValueError 
1272          data, pde_args, plotdata, ssorted, colors, \
 
1273                csorted, ixdata, sstats = loadObjects(pde_name) 
1274          print "Loaded data set and stats for %s"%a.name 
1275          # don't check the data field
 
1276          if filteredDict(a, ['data','bin_width'], neg=True) != \
 
1277                   filteredDict(pde_args, ['data','bin_width'], neg=True): 
1278              raise "Incompatible argument struct from file %s"%pde_name 
1279          if a.data is None: 
1280              a.data = data 
1281          elif not all(a.data == data): 
1282              raise "Incompatible data set loaded from file %s"%pde_name 
1283      except ValueError: 
1284          if a.data is None: 
1285              try: 
1286                  data = eval(a.data_gen_str, globals(), a.data_gen_fun) 
1287              except: 
1288                  print "Problem re-calculating data from given information" 
1289          else: 
1290              data = a.data 
1291          print "Recalculating PD-E for %s"%a.name 
1292          try: 
1293              csource=a.color_source 
1294          except AttributeError: 
1295              csource=None 
1296          if verbose: 
1297              scatter_fig = None 
1298          else: 
1299              scatter_fig = 0 
1300          if a.data is None: 
1301              a.data = data 
1302          plotdata, ssorted, colors, csorted, ixdata, sstats, handle = \
 
1303                    scatterplot_slopes(data,
 
1304                              startix=a.startix, stopix=a.stopix,
 
1305                              num_samples=a.num_samples,
 
1306                              Delta=a.Delta, color_source=csource, step=a.step,
 
1307                              maxD=a.maxD, fignum=scatter_fig) 
1308          saveObjects([data, a, plotdata, ssorted, colors, csorted, ixdata, sstats],
 
1309                      pde_name, force=True) 
1310      if verbose: 
1311          scatter_histo(plotdata,bin_width=a.bin_width,select=0) 
1312          scatter_histo(plotdata,bin_width=a.bin_width,select=1) 
1313          info(sstats) 
1314      if saveplot: 
1315          save_info='result_'+a.name 
1316      else: 
1317          save_info='' 
1318      if 'show_seq' in a.keys(): 
1319          show_seq = a['show_seq'] 
1320      else: 
1321          show_seq = False 
1322      more_info=prep_secant_figure(data, plotdata, ssorted, colors, ixdata, sstats,
 
1323                     a.startix, a.stopix, a.Delta, secfig, save=save_info,
 
1324                     show_seq=show_seq) 
1325      try: 
1326          more_info['handle']=handle 
1327      except UnboundLocalError: 
1328          more_info['handle']=None 
1329      more_info['stats']=sstats 
1330      more_info['args']=a 
1331      return more_info

1332
```

  


| Home | Trees | Indices | Help | PyDSTool |
| --- | --- | --- | --- | --- |

|  |  |
| --- | --- |
| Generated by Epydoc 3.0.1 on Fri May 4 15:24:26 2012 | http://epydoc.sourceforge.net |
